# Allelic, Genotypic, and Haplotypic Analysis of Cytokine IL17A, IL17F, and Toll-like Receptor TLR4 Gene Polymorphisms in Metabolic-Dysfunction-Associated Steatotic Liver Disease: Insights from an Exploratory Study

**DOI:** 10.3390/life14101327

**Published:** 2024-10-18

**Authors:** Sorina-Cezara Coste, Olga Hilda Orășan, Angela Cozma, Vasile Negrean, Teodora Gabriela Alexescu, Mirela Georgiana Perne, George Ciulei, Adriana Corina Hangan, Roxana Liana Lucaciu, Mihaela Iancu, Lucia-Maria Procopciuc

**Affiliations:** 1 4th Department of Internal Medicine, Faculty of Medicine, “Iuliu Hațieganu” University of Medicine and Pharmacy, 400012 Cluj-Napoca, Romania; secara.sorina@umfcluj.ro (S.-C.C.); hilda.orasan@umfcluj.ro (O.H.O.); angelacozma@umfcluj.ro (A.C.); vasile.negrean@umfcluj.ro (V.N.); teodora.alexescu@umfcluj.ro (T.G.A.); alb.mirela@umfcluj.ro (M.G.P.); george.ciulei@umfcluj.ro (G.C.); 2 Department of Inorganic Chemistry, Faculty of Pharmacy, “Iuliu-Hațieganu” University of Medicine and Pharmacy, 400012 Cluj-Napoca, Romania; adriana.hangan@umfcluj.ro; 3 Department of Pharmaceutical Biochemistry and Clinical Laboratory, Faculty of Pharmacy, “Iuliu-Hațieganu” University of Medicine and Pharmacy, 400012 Cluj-Napoca, Romania; liana.lucaciu@umfcluj.ro; 4 11th Department of Medical Education, Medical Informatics and Biostatistics, “Iuliu Hațieganu” University of Medicine and Pharmacy, 400012 Cluj-Napoca, Romania; 5 Department of Molecular Sciences, Faculty of Medicine, “Iuliu Hațieganu” University of Medicine and Pharmacy, 400349 Cluj-Napoca, Romania; lprocopciuc@umfcluj.ro

**Keywords:** MASLD, interleukin 17, toll-like receptor 4, IL17F-A7488G, IL17A-G197A, TLR4-Asp299Gly, TLR4-Thr399Ile

## Abstract

(1) Background: Interleukin 17 (IL17) and toll-like receptor 4 (TLR4) elevate the risk of metabolic and liver diseases. (2) Methods: This study’s objective was to explore the association of IL17 and TLR4 gene polymorphisms with MASLD susceptibility and test their effect on serum IL17 and TLR4 levels. A total of 43 patients with MASLD (MASH/MAFL) and 38 healthy individuals were genotyped for IL17F-*A7488G*, IL17A-*G197A*, TLR4-*Asp299Gly*, and TLR4-*Thr399Ile* polymorphisms using PCR-RFLP. ELISA methods determined IL17F, IL17A, and TLR4 serum levels. (3) Conclusions: Patients carrying the variant genotypes (*A/G + G/G*) of IL17-*A7448G* (OR = 5.25), (*G/A + A/A*) of *IL17-G197A* (OR = 10.57), (*Asp/Gly + Gly/Gly*) of TLR4-*Asp299Gly* (OR = 3.52), or (*Thr/Ile + Ile/Ile*) of TLR4-*Thr399Ile* (OR = 9.87) had significantly increased odds of MASH. Genotype (*G/A + A/A*) of IL17-*G197A* was significantly associated with the odds of MAFL (*p* = 0.0166). Allele *A* of the *IL17-G197A* polymorphism was significantly related to increased odds of MAFL (OR = 4.13, *p* = 0.0133). In contrast, allele *A* of IL17-*G197A* (OR = 5.41, *p* = 0.008), allele *Gly* of TLR4-*Asp299Gly* (OR = 3.19, *p* = 0.046), and allele *Ile* of TLR4-*Thr399Ile* (OR = 6.94, *p* = 0.008) polymorphisms were significantly related to an increased risk of MASH. Allele *A* of IL17A-*G197A*, allele *Gly* of TLR4-*Asp299Gly*, and allele *Ile* of TLR4-*Thr399Ile* gene polymorphisms were significantly associated with the increased odds of MASLD. In patients with MASLD, we found significant influence from the IL17A-*G197A* gene polymorphism on IL17F levels (*p* = 0.0343).

## 1. Introduction

Metabolic-dysfunction-associated steatotic liver disease (MASLD), previously referred to as non-alcoholic fatty liver disease (NAFLD), ref. [1] is the most prevalent chronic liver condition globally, with incidence rates continuing to rise. In Europe, the prevalence is around 25–26%, while in the Romanian population, it is approximately 20% [2]. MASLD is now recognized as the hepatic manifestation of metabolic syndrome (MS), a cluster of conditions including obesity, hypertension, dyslipidemia, and insulin resistance [3]. MS is a significant risk factor for liver fibrosis and cirrhosis, making MASLD an increasingly important public health concern [2]. A large cohort analysis by Lonardo et al. (2015) included over 1000 patients and established a clear correlation between MS components, such as obesity, insulin resistance, and dyslipidemia, and the prevalence and severity of MASLD. This study underscores that MS is a key risk factor for MASLD development, contributing to liver inflammation and fibrosis progression [4].

Toll-like receptors (TLRs) are proteins in the immune system that recognize and respond to pathogens. TLR4, for example, detects lipopolysaccharide (LPS) and triggers the release of various cytokines. It is expressed in cell types such as hepatocytes and monocytes [5]. TLR4 is a crucial link between gut microbiota, endotoxemia, and liver injury [6]. Sharifnia T et al. (2015) found increased TLR4 expression in MASH patients compared to those with simple steatosis [7]. Soluble TLR4 (sTLR4), a form of TLR4 that circulates in the body, works as a decoy receptor by binding to its ligands, reducing TLR4 signaling activation. This process has been found to control inflammation. Higher levels of sTLR4 seem to have a protective effect against the long-term inflammation seen in MASLD. Research indicates that sTLR4 could be a valuable biomarker for tracking liver disease progression [8].

The mechanisms driving the progression from simple steatosis to fibrosis in MASLD remain unclear. Th17 cells, a subset of helper T cells, produce IL17 and are crucial in host defense against bacterial and fungal pathogens.

IL-17A and IL-17F play an essential role in liver inflammation, fibrogenesis, and the progression of carcinogenesis in various liver diseases, such as hepatitis B and C virus infections (HBV/HCV), non-alcoholic steatohepatitis (NASH), alcoholic liver disease (ALD), hepatocellular carcinoma (HCC), and chronic toxic liver injury. In the case of HBV/HCV infections, IL-17A/F is increased and promotes chronic inflammation and fibrosis by recruiting immune cells and stimulating proinflammatory cytokines [9]. In NASH, these cytokines induce inflammation and fibrosis by activating hepatic stellate cells and worsening insulin resistance, contributing to metabolic dysfunction [10]. In ALD, IL-17A/F amplifies liver damage and fibrosis, increasing immune responses and intestinal permeability, thereby intensifying inflammation [11]. In HCC, IL-17A/F contributes to tumor genesis, angiogenesis, and immune evasion in chronic inflammation [12]. In chronic toxic liver injury, IL-17A/F intensify injury and fibrosis through their proinflammatory and pro-fibrotic effects [11].

Tang et al. (2011) investigated the role of Th17 cells in a MASLD mouse model and discovered that Th17 cells and IL17 were associated with hepatic steatosis and proinflammatory response in MASLD, facilitating the transition from simple steatosis to steatohepatitis [13].

The TLR4 gene polymorphisms *Asp299Gly* and *Thr399Ile* represent specific genetic variations within the TLR4 gene (9q32-33), responsible for encoding the TLR4 protein. One of these variations is a nonsynonymous single nucleotide polymorphism (SNP) situated in the gene region encoding the extracellular domain of TLR4 [14]. This variation is a +986A/G point mutation, resulting in the substitution of aspartic acid (Asp) with glycine (Gly) at the 299th position of the protein (*Asp299Gly*-rs 4986790). This polymorphism is linked to altered immune and inflammatory responses. The other polymorphism in the TLR4 gene is a +1196C/T point mutation, which leads to the replacement of threonine with isoleucine at the 399th position (*Thr399Ile*-rs4986791). Like the *Asp299Gly* variant, the *Thr399Ile* variant can alter the receptor’s pathogen response, potentially affecting the severity and outcome of infectious diseases and inflammatory responses [15].

IL17A-*G197A* (rs2275913) and IL17F-*A7488G* (rs763780) are specific gene polymorphisms associated with the IL17 family of cytokines, which play a crucial role in immune and inflammatory responses. The IL17A-*G197A* polymorphism occurs in the gene coding for the IL17A cytokine (chr 6p12.2), signifying an SNP where guanine (G) is substituted by adenine (A) at position 197 in the DNA sequence. Such changes can increase the production levels of IL17A. IL17A is vital for pathogen defense, but elevated levels are associated with various inflammatory and autoimmune conditions, including psoriasis, rheumatoid arthritis, and MASLD [16]. Similarly, the IL17F-*A7488G* polymorphism involves a substitution of adenine (A) with guanine (G) at position 7488 in the IL17F gene (chr 6p12.2). IL17F is closely related to IL17A, sharing similar roles in immune response and inflammation. Variations in the IL17F gene can affect the cytokine’s production and activity, potentially impacting inflammatory processes and disease susceptibility [17].

The link between TLR4 and IL17A/F has been studied due to their essential role in the immune and inflammatory response associated with various pathologies. TLR4 represents a crucial component of the innate immune system, while IL17A and IL17F, members of the IL17 cytokine family, are key factors in promoting inflammatory and autoimmune responses. Genetic research has suggested a significant link between TLR4 and IL17A/F. For example, the study by Wang et al. (2016) on pulmonary tuberculosis showed that polymorphisms in genes TLR4 and IL17A/F influence susceptibility to the disease, highlighting the genetic interactions between these molecules [18]. In addition, the immune response in arterial hypertension, modulated by TLR4 and IL17A, studied by Simundic et al. (2017) further supports their interconnected roles in chronic inflammatory conditions [19].

Because of the pro-inflammatory role of IL17 and TLR4, the objectives of the current study were to explore the associations between (i) IL17 (*A7448G* and *G197A*) and TLR4 (*Asp299Gly* and *Thr399Ile*) gene polymorphisms and MASLD susceptibility; (ii) IL17 (*A7448G* and *G197A*) and TLR4 (*Asp299Gly* and *Thr399Ile*) gene polymorphisms and MASH/MAFL susceptibility at the allelic, genotypic, and haplotypic level; and (iii) to test the effect of the studied gene polymorphisms on plasmatic levels of IL17 and TLR4 in MASLD patients as well as in controls.

## 2. Materials and Methods

The current study was a prospective, longitudinal, case-cohort study conducted at the CF Clinical University Hospital in Cluj-Napoca, Romania, between October 2018 and August 2022. The study was approved by the Medical Ethics Committee of the “Iuliu Hatieganu” University of Medicine and Pharmacy, Cluj-Napoca (no 322/26 July 2018). It was conducted in accordance with the Declaration of Helsinki. Prior to enrollment, each patient provided written informed consent. The cohort included 43 patients with MS and NAFLD (either NASH or NAFL) and 38 healthy individuals. The inclusion criteria for patients were as follows: 1—the presence of metabolic syndrome (MS) and 2—the presence of hepatic steatosis. The diagnosis of MS was established if at least three of the following five criteria were present: a. hyperglycemia: fasting glucose level values ≥100 mg/dL or medication for elevated glucose; b. low HDL-cholesterol values: <40 mg/dL in men and <50 mg/dL in women or treatment in the case of low HDL-C; c. hypertriglyceridemia: TG ≥ 150 mg/dL or taking medication for elevated TG; d. waist circumference ≥102 cm in men and ≥88 cm in women; e. hypertension defined as BP ≥ 130/85 mmHg or the presence of antihypertensive treatment.

The presence of NAFLD was based on the following criteria: a. diagnosis of hepatic steatosis by imaging methods (abdominal ultrasound or anatomopathological examination of fragment of liver tissue extracted by liver biopsy that evaluates the degree of hepatic steatosis, degree of necroinflammatory activity and stage of liver fibrosis); b. exclusion of alcohol consumption, defined as >30 g/day for men and >20 g/day for women; c. evaluation of serum transaminase levels to support the diagnosis of steatohepatitis. The exclusion criteria were as follows: 1. refusal to participate in the study; 2. the presence of comorbidities based on medical history that is associated with the presence of hepatic steatosis: alcoholic hepatitis, viral hepatitis B and C, Wilson’s disease, autoimmune hepatitis, hemochromatosis, consumption of drugs (amiodarone, methotrexate, glucocorticoids, valproate, tamoxifen); 3. the presence of conditions based on medical history that could influence genetic results on the presence of polymorphisms of IL17 and TLR4 genes: asthma, rheumatoid arthritis, viral hepatitis B or C, inflammatory bowel disease. Healthy control subjects were recruited from individuals seeking a routine health check-up at the CF Clinical University Hospital in Cluj-Napoca, Romania, who showed no evidence of hepatic steatosis at abdominal ultrasound. The controls had a similar distribution of age, gender, and ethnicity as the case cohort.

### 2.1. Diagnosis of Hepatic Steatosis and Liver Biopsy

The ultrasound diagnosis of liver steatosis was based on a comparative assessment of liver and kidney echogenicity, as recommended by the Practice Guidelines of the American Gastroenterology Association [20]. US-guided liver biopsy was performed for patients diagnosed with hepatic steatosis via ultrasound. Liver biopsy specimens (at least 1.6 cm long and 5 μm thick) were stained with hematoxylin/eosin, Masson’s trichrome, and PAS and examined by an anatomopathologist. The pathological diagnosis of NAFLD requires the presence of the following features: 1. the histological findings of liver biopsy are consistent with the pathological diagnosis criteria of fatty liver disease; 2. the NAFLD activity score (NAS) is assessed routinely to make a pathological diagnosis according to Kleiner et al.’s NAS scoring system, according to which patients with NAS < 3 were considered as not having NASH and those with scores between 3 and 5 were diagnosed as probably having NASH; 3. there is no history of alcohol consumption as mentioned above in the 12 months prior the study.

### 2.2. Clinical and Biochemical Measurements

We recorded general data from each subject: age, sex, residence, body mass index (BMI calculated weight in kilograms (kg) divided by height in meters (m) squared), waist circumference (WC), systolic and diastolic blood pressure (SBP, DBP) values, status of smoking, and other comorbidities. A blood sample was obtained from each patient for routine assessments: fasting plasma glucose (FPG), total cholesterol (TC), high-density lipoprotein cholesterol (HDL-C), triglyceride (TG), aspartate aminotransferase (AST), alanine aminotransferase (ALT), alkaline phosphatase (ALP), gamma-glutamyl transferase (GGT), platelets count (PLT), serum bilirubin, erythrocyte sedimentation rate (ESR), and C reactive protein (CRP).

### 2.3. Serum IL17A/F and TLR4 Determination

We determined human IL17A/F and TLR4 concentrations in serum using micro enzyme-linked immunosorbent assay (ELISA) plates pre-coated with an antibody specific to human IL17A/F (BioVendor Laboratorni medicina a.s.; Elabscience Biotechnology Inc., Houston, TX, USA) and TLR4 (Elabscience Biotechnology Inc., Houston, TX, USA) according to the manufacturer’s protocol (IL7A-Catalog No: E-EL-H0105; IL 17F-Catalog No: RAF043R and TLR4-Catalog No: E-EL-H6123). We spectrophotometrically measured the optical density (OD) at 450 nm using a microplate reader (Absorbance Microplate Reader Sunrise Tecan; Tecan Group Ltd., Männedorf, Switzerland) and Biochrom Asys Atlantis Microplate Washer (Biochrom Ltd., Cambridge, UK).

### 2.4. Genetic Analysis

#### 2.4.1. DNA Extraction

Five milliliters of peripheral blood samples were collected from patients and controls in EDTA tubes. High-molecular-weight DNA was extracted using a Zymo research kit (Quick-DNAMiniprep, Kit-Zymo Research Corporation, Freiburg, Germany). The DNA samples were stored at −20 °C until polymerase chain reaction–restriction fragment length polymorphism (PCR-RFLP) methods were used.

#### 2.4.2. PCR-RFLP Methods

**Figure 1:**
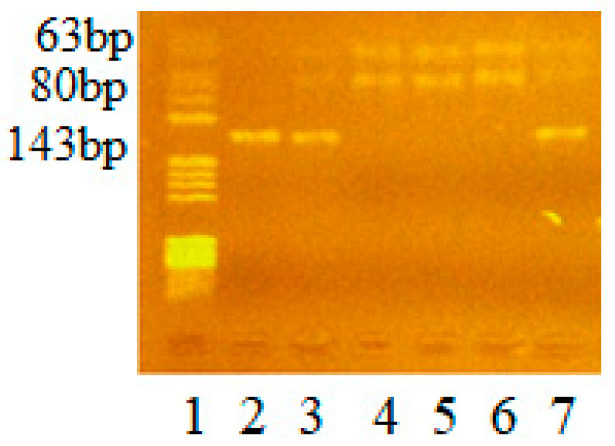
Identification of IL17F-*A7488G* polymorphism. Lane 1—pBRHaeIII Digest DNA molecular marker V; lane 2—negative genotype GG7488-fragment of 143 bp; lanes 3, 7—heterozygous genotype GA7488-fragments of 143 bp, 80 bp, 63 bp; lanes 4, 5, 6—homozygous genotype AA-fragments of 80 bp, 63 bp.

**Figure 2:**
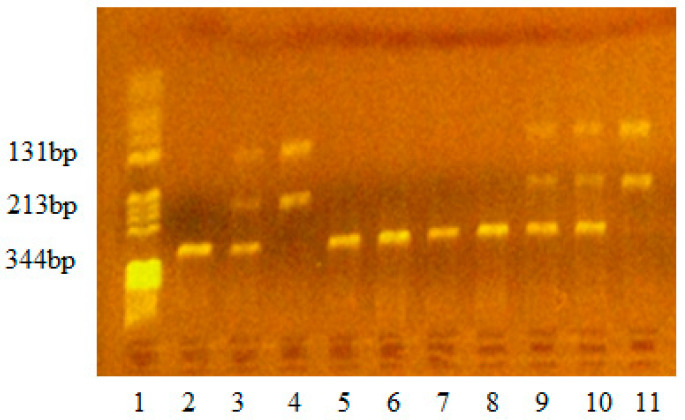
Identification of IL17A-*G197A* polymorphism. Lane 1—pBRHaeIII Digest DNA molecular marker V; lane 2, 5, 6, 7, 8—negative genotype GG197-fragment of 344 bp; lanes 3, 9, 10—heterozygous genotype GA197-fragments of 344 bp, 213 bp, 131 bp; lanes 4, 11—homozygous genotype AA197-fragments of 213 bp, 131 bp.

**Figure 3:**
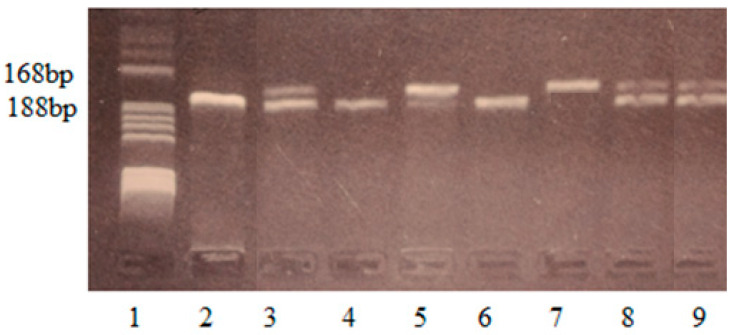
Identification of TLR4-*Asp299Gly* polymorphism. Lane 1—pBRHaeIII Digest DNA molecular marker V; lane 2, 4, 6—negative genotype Asp/Asp299-fragment of 188 bp; lanes 3, 5, 8, 9—heterozygous genotype Asp/Gly299-fragments of 188 bp, 168 bp; lane 7—homozygous genotype Gly/Gly299-fragments of 168 bp.

**Figure 4:**
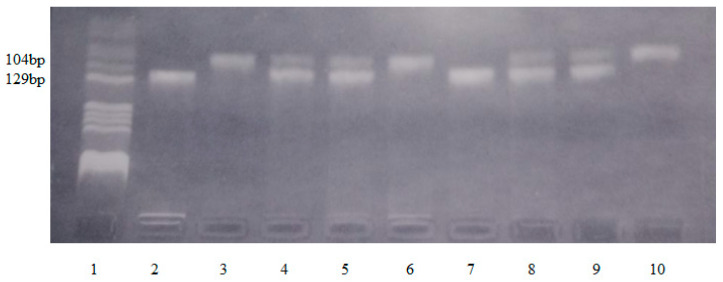
Identification of TLR4-*Thr399Ile* polymorphism. Lane 1—pBRHaeIII Digest DNA molecular marker V; lanes 2, 7—negative genotype Thr/Thr399-fragment of 129 bp; lanes 3, 6, 10—homozygous genotype Thr/Ile399-fragment of 104 bp; lanes 4, 5, 8, 9—heterozygous genotype Ile/Ile399-fragments of 124 bp, 104 bp.

In the present study, genotyping of the IL17F-*A7488G*, IL17A-*G197A*, TLR4-*Asp299Gly*, and TLR4-*Thr399Ile* polymorphisms was performed on DNA samples by polymerase chain reaction (PCR) and restriction fragment length polymorphism analysis (RFLP) using the methods described by Wu et al. (2010), Dhifallah et al. (2009), and Bahadori et al. (2014) and optimized in our laboratory [21,22,23]. Genomic DNA was amplified in a BIORad cycler (Bio-Rad Life Science, Hercules, CA, USA). The reaction was carried out in a 25 μL mixture which contained 5 μL DNA template, 0.2 μm primers, 200 μm deoxynucleotide triphosphates (dNTPs), PCR buffer containing 50 mM KCl, 10 mM Tris-HCl (pH 8.3), 2.0 mM MgCl_2_, and 0.625 units FIREPol DNA polymerase. The primers were reconstituted in sterile distilled water (100 μm solution) and stored at −20 °C. The specificity of the PCR amplification was checked by electrophoresis of 5 µL amplified PCR products on 2% agarose gel stained with 10 mg/mL ethidium bromide solution. The gel was visualized on a UV transilluminator. The PCR amplicons had 143 bp (IL17F-*A7488G*), 344 bp (IL17A-*G197A*), 188 bp (TLR4-*Asp299Gly*), and 124 bp (TLR4-*Thr399Ile*). After PCR amplification, 6 μL amplicons were digested with 2U of specific restriction enzyme for 3 h at 37 °C. The specificity of the RFLP method was checked by electrophoresis on a 3% agarose gel stained with 10 mg/mL ethidium bromide solution. The gel was visualized on a UV transilluminator. The FIREPol DNA polymerase was from Solis BioDyne (Solis BioDyne OÜ, Teaduspargi, Tartu, Estonia), the primers were from Invitrogen Thermo Fisher Scientific (Waltham, MA, USA), and the restriction enzyme was from New England Biolabs (New England Biolabs UK, Ltd., Hitchin, UK). Details of PCR-RFLP genotyping conditions have been provided in Table 1 and Figure 1, Figure 2, Figure 3 and Figure 4.

### 2.5. Statistical Analysis

Demographic, anthropometric, laboratory, and histological features were summarized using an arithmetic mean and standard deviation for variables with Gaussian distribution or median with Interquartile Range (IQR = (Q1, Q3), Q1 = first quartile; Q3 = third quartile) for variables with deviations from the Gaussian distribution or absolute and relative frequency (%) for qualitative nominal variables. Univariate Gaussian distribution was assessed using different statistical methods (quantile–quantile plot and Shapiro–Wilk test with Holm correction for multiple comparisons).

Comparisons of demographic, anthropometric, and laboratory characteristics between control, MAFL, and MASH groups were performed using ANOVA, Welch’s ANOVA test, Kruskal–Wallis test, Chi-squared test, and exact Fisher’s test. Post hoc analysis using the Tukey HSD test or Dunn’s test was conducted in the case of statistically significant results. Differences in allele and genotype frequencies of the studied gene polymorphisms between control, MAFL, and MASH groups were compared using the Chi-square test or exact Fisher’s test. The departure from Hardy–Weinberg Equilibrium (HWE) for studied SNPs was tested using the exact Chi-square test from the “genetics” R package [24].

The associations between the studied SNPs and odds of MAFL and MASH were evaluated by unconditional multinomial logistic regression. Because of the small number of cases, we merged MAFL and MASH cases into a single category named MASLD and evaluated the odds of MASLD using binomial logistic regression models. The effect size of the association was described using the unadjusted odds ratio (OR) with a 95% confidence interval (95% CI). Multinomial and binomial logistic regression analysis was performed under three different (codominant, dominant, and recessive) inheritance genetic models using the R-project package “nnet” [25,26].

Haplotype analysis was performed in order to estimate haplotype frequencies using the expectation-maximization algorithm of the R-project package “haplo.stats” [27]. The effect size of the association was estimated using OR and 95% CI, with the most frequent haplotype as the reference haplotype. All statistical analysis was performed in R software, version 4.4.0 [28].

The results of two-tailed statistical tests were considered statistically significant at an estimated significance level of *p* < 0.05. In addition, to account for multiple comparisons, we also reported corrected *p*-values using the Benjamini–Hochberg method.

## 3. Results

A summary of the patients’ demographic, anthropometric, laboratory, and clinical characteristics is provided in Table 2. We found no significant difference in mean age between controls, MAFL, and MASH patients (*p* = 0.584). Body mass index was significantly higher in patients with MAFL compared to controls (adjusted *p* = 0.002, mean difference BMI: 4.5, 95% CI: 1.4–7.6) and patients with MASH compared to controls (adjusted *p* = 0.0002, mean difference BMI: 5.1, 95% CI: 2.2–8.0). Similar results were noticed for waist circumference (MAFL patients vs. controls: adjusted *p* = 0.021, mean difference: 10.0, 95% CI: 1.3–18.7, MASH patients vs. controls: adjusted *p* = 0.001, mean difference: 13.0, 95% CI: 4.8–21.2). We found no significant difference between mean BMI and WC between MAFL and MASH patients (Tukey HSD test, adjusted *p* > 0.05). FPG, T-cholesterol, TG, ALT, ALP, and GGT levels significantly differed between control, MAFL, and MASH patients (Table 2). Post hoc analysis revealed that significant differences were due to the differences between the control group and MASH group for all laboratory parameters: Dunn test, adjusted *p* = 0.003 for glycemia, Tukey HSD test, adjusted *p* = 0.0141 for cholesterol total, Dunn test, adjusted *p* = 0.0002 for triglycerides, Dunn test, adjusted *p* = 0.0074 for ALT, Tukey HSD test, adjusted *p* = 0.0138 for ALP, Dunn test, adjusted *p* = 0.0028 for GGT. The only exception was FPG, where we found a significant difference between MAFL and MASH groups (Dunn test, adjusted *p* = 0.0188). In addition, we found a significant difference in the relative frequency of T2DM, dyslipidemia, obesity, CHD, and PA in control, MAFL, and MASH patients (Table 2).

### 3.1. Genetic and Allelic Association of IL17 (A7488G, G197A) and TLR4 (Asp299Gly, Thr399Ile) Gene Polymorphisms with Odds of MAFL and Odds of MASH

The genotypes distribution of IL17F-A7448G and TLR4-Asp299Gly gene polymorphisms conformed to the HWE in the control group (*p* = 0.0795, *p* = 1.000) but IL17A-G197A and TLR4-Thr399Ile gene polymorphisms showed a significant deviation from the HWE (*p* = 0.0186, *p* = 0.040). Chi-square or Fisher’s exact association tests were used to assess the genotype frequency distributions of the studied gene polymorphisms stratified by controls and types of MASLD (MAFL and MASH) (Table 3). All variant genotypes of the studied IL17 and TLR4 gene polymorphisms had a higher frequency in MAFL and MASH patients than in controls (Table 3).

Results of univariable multinomial logistic regression models applied to the dominant inheritance genetic model indicated a significant association between all variant genotypes of the studied gene polymorphisms and MASH susceptibility (Table 4). Patients with MASH were significantly more likely to have a variant genotype (*A/G + G/G*) of IL17-*A7448G* gene polymorphism (corrected *p* = 0.0338), variant genotype (*G/A + A/A*) of IL17-*G197A* gene polymorphism (corrected *p* = 0.0008), variant genotype (*Asp/Gly + Gly/Gly*) of TLR4-*Asp299Gly* gene polymorphism (corrected *p* = 0.0338), or variant genotype (*Thr/Ile + Ile/Ile*) of TLR4-*Thr399Ile* gene polymorphism (corrected *p* = 0.0138).

Results from allelic analysis indicated that the allele *A* of the IL17-*G197A* gene polymorphism was significantly related to an increased risk of MAFL (corrected *p* = 0.0133), while allele A of the IL17-*G197A* gene polymorphism (corrected *p* = 0.008), allele *Gly* of the TLR4-*Asp299Gly* gene polymorphism (corrected *p* = 0.046), and allele *Ile* of the TLR4-*Thr399Ile* gene polymorphism (corrected *p* = 0.008) were significantly related to an increased risk of MASH.

### 3.2. Genetic and Allelic Association of IL17 and TLR4 Gene Polymorphisms with Odds of MASLD

The distributions of both genotypes and alleles for IL17F-A7488G, IL17A-G197A, TLR4-Asp299Gly, and TLR4-Thr399Ile were significantly different in MASLD patients and controls (Table 4). From allelic analysis, allele A of IL17A-G197A, allele Gly of TLR4-Asp299Gly, and allele Ile of TLR4-Thr399Ile gene polymorphisms were significantly related to the increased odds of MASLD (Table 5).

### 3.3. Haplotype Association of IL17 and TLR4 Gene Polymorphisms with the Odds of MASLD

The IL17 and TLR4 gene polymorphisms were in weak to moderate pairwise linkage disequilibrium in MASLD group: D′_IL17-A748/-G197_ = 0.39, *p* = 0.3710, D′_TLR4-Asp/-thr_ = 0.19, *p* = 0.1793; meanwhile, in the control group, we found a strong pairwise linkage disequilibrium: D′_IL17-A748/-G197_ = 0.99, *p* = 0.5243, D′_TLR4-Asp/-thr_ = 0.98, *p* = 0.5873.

The results of haplotype analysis showed that the A/A two-locus haplotype of IL17 gene polymorphisms was a significant risk factor for MASLD, while none of the most frequent two-locus haplotypes of TLR4 were significantly associated with an increased risk of MASLD (Table 6).

### 3.4. Serum IL17A, IL17F, and TLR4 Levels in MASLD Patients Compared to Controls and Possible Associations with IL17A, IL17F, and TLR4 Polymorphisms

Serum levels of IL17A were distributed significantly differently between MASLD patients and controls (Mann–Whitney U test, *p* = 0.00696), with patients with MASLD having higher values of IL17 compared to controls (median = 108.9, IQR: 90.1–214.7 vs. median = 76.2, IQR: 42.9–115.8). Serum levels of IL17F were distributed significantly differently between MASLD patients and controls (Mann–Whitney U test, *p* = 0.0080), with patients with MASLD having higher values of IL17 compared to controls (median = 15.2, IQR: 7.5–26.9 vs. median = 7.3, IQR: 5.3–12.4). Serum levels of TLR4 were distributed significantly differently between MASLD patients and controls (Mann–Whitney U test, *p* < 0.0001), with patients with MASLD having higher values of IL17 compared to controls (median = 1820.5, IQR: 1482.4–1963.7 vs. median = 1296.6, IQR: 983.4–1460.6).

The serum levels of IL17A were significantly different in controls with genotype A/A compared to those with genotype A/G + G/G of IL17F-A7448G gene polymorphism, while in the MASLD group, we found no significant association between IL17F-A7448G polymorphism and serum levels of IL17A. The serum levels of IL17F were significantly different in controls with genotype Asp/Asp compared to those with genotype Asp/Gly + Gly/Gly of the TLR4-Asp299Gly gene polymorphism while, in the MASLD group, we found no significant association between the TLR4-Asp299Gly polymorphism and serum levels of IL17F but we found a significant association with the IL17A-G197A gene polymorphism (Table 7).

## 4. Discussion

In this study, we explore IL17 (*A7448G* and *G197A*) and toll-like receptor TLR4 (*Asp299Gly* and *Thr399Ile*) gene polymorphisms in patients with simple steatosis (MAFL) or steatohepatitis (MASH) and MASLD. We also wanted to test the effect of the studied gene polymorphisms on the plasmatic levels of IL17 and TLR4 in MASLD patients and controls.

Toll-like receptors (TLRs) are members of the interleukin 1 receptor family. They play an essential role in activating innate immune responses by recognizing molecular patterns associated with conserved pathogens such as lipopolysaccharides (LPS). Genetic polymorphism in the TLR4 gene could influence metabolic syndrome (MS) susceptibility. The origin of the polymorphisms of toll-like receptor 4 (TLR4), +986A/G (*Asp299Gly*), and +1196C/T (*Thr399Ile*) investigated in our study is considered to be rooted in Africa due to the selective pressure exerted by malaria. The *Asp299Gly* polymorphism appeared first, followed by *Thr399Ile*, both associated with changes in the receptor ligand-binding site. These polymorphisms have a varied prevalence in different populations, having higher frequencies in Indo-European populations and being almost absent in Polynesian and native South American populations. In contrast, the TLR4 *+3725G/C* and *+11367G/C* polymorphisms identified in the Han Chinese population with type 2 diabetes mellitus (T2DM) appear as protective factors, significantly reducing the risk of T2DM. These polymorphisms can influence gene expression through posttranscriptional regulatory mechanisms. The study did not find the *+986A/G* (*Asp299Gly*) and *+1196C/T* (*Thr399Ile*) polymorphisms in the Han Chinese population, suggesting their geographical and ethnic specificity [29].

According to different studies, polymorphisms of the TLR4 gene significantly influence inflammatory mechanisms and metabolic pathways, affecting inflammation modulation, insulin resistance, and lipid metabolism. These processes contribute to the progression of MS and are involved in developing MASLD. However, the role of these polymorphisms in the risk of MS is still controversial. A study that looked at haplotypes showed that the *Asp299Gly* and *Thr399Ile* polymorphisms could influence the risk of type 2 diabetes (T2D) in patients with high levels of triglycerides (TG) and HDL-cholesterol (HDL-C) [30]. On the other hand, research by Illig et al. (2003) and Maldonado-Bernal et al. (2011) found no association between *Asp299Gly* and T2D or MS components [31,32]. Cuda et al. (2011) reported that the TLR4-*Asp299Gly* polymorphism is associated with increased insulin resistance and alters the relationship between dietary saturated fat and HDL-C. Their study showed that carriers of the *Gly* allele have higher insulin levels and higher HOMA-IR and HOMA-β indices, suggesting lower insulin sensitivity than carriers of the *Asp* allele [33]. In another study, deletion of the TLR4 gene in mice attenuated hypercholesterolemia and hyperglycemia, suggesting that TLR4 could be a target for preventing MS [34]. Penas-Steinhardt et al. (2010) pointed out that the *Asp299Gly* polymorphism is associated with higher risks of MS, lipid accumulation syndromes, such as MASLD, and insulin resistance. They found that carriers of the Asp/Asp genotype have a 2.03 (95% CI, 1.02–4.05, *p* = 0.04) higher risk of MS. Furthermore, patients with higher triglyceride (TG) levels and lower high-density lipoprotein (HDL) levels have a 4.43 times greater risk of developing metabolic syndrome (MS) compared to those with normal lipid profiles [35].

It is well known that MS is considered a risk factor for NAFLD. Research on the relationship between TLR4 gene polymorphisms NAFLD associated with MS highlights the significant role of genetic variations in TLR4 and inflammation in the liver. According to a study conducted on TLR4 polymorphisms, specifically *Asp299Gly* and *Thr399Ile*, Kiziltas et al. (2014) found associations with NAFLD and their influence on susceptibility to the disease. The study found a higher frequency of the *Asp299Gly* polymorphism in a homozygous form in patients with NAFLD (5.9%) compared to the control group (2.5%). However, they also found a higher frequency of heterozygous carriers in the control group compared to NAFLD patients, suggesting the protective role against NAFLD of this polymorphism in the heterozygous form [36]. Furthermore, Nguyen et al. (2018) pointed out that a high expression of TLR4 is associated with the occurrence of NAFLD-related hepatocellular carcinoma (HCC), further emphasizing its role in NAFLD progression [37].

IL17 polymorphisms play a significant role in the pathogenesis of MASLD by contributing to the inflammatory process. IL17, produced mainly by CD4+ (TH17) and CD8+ (Tc17) T cells, is essential in mediating inflammation and liver fibrosis. Increased levels of IL17 are associated with progression from steatosis to steatohepatitis, cirrhosis, and even hepatocarcinoma [38]. Specific genetic polymorphisms (SNPs) in IL17A/F, especially *G7488A* and *G197A*, have been correlated with the modulation of systemic inflammation and fibrosis in NAFLD, influencing genetic susceptibility to MASLD [39]. The haplotypes G7488A and G197A are important in the context of MASLD and its progressive form, MASH, due to their potential roles in genetic susceptibility and disease progression. Genetic variations in mitochondrial DNA, such as G7488A, influence mitochondrial function, which is crucial in liver metabolism. Mitochondrial dysfunction has been strongly linked to oxidative stress and impaired fatty acid oxidation, both of which are central to the pathophysiology of MASLD/MASH. Variations like G197A could also affect liver function by influencing the regulatory genes involved in lipid metabolism and inflammation, key pathways in MASLD progression. These haplotypes may modulate how the liver responds to metabolic stress, contributing to varying disease severity among individuals.

To the best of our knowledge, no articles have been published regarding the relationship between IL 17A/F and MASLD so far. Our study is among the first prospective studies to evaluate a potential link between IL 17A/F gene polymorphisms and MASLD in a Romanian patient population.

Studies on the relationship between IL17 and MASLD polymorphisms suggest that IL17 has a significant role in the pathogenesis and progression of the disease. One example is Olveira et al.’s study (2023), which described the critical role of IL17 as a pathogenetic link between psoriasis and MASLD, highlighting the involvement of IL17 in systemic inflammation and liver fibrosis [16].

In our study, patients carrying the variant genotype of IL17A-*G197A* gene polymorphism had significantly higher odds of MAFL. Similar to our results, Akbulut et al. (2019) found that the IL 17A-*G197A* polymorphism is associated with the development of MASLD in obese Turkish children ([OR] 2.05, 95% confidence interval: 1.12–3.77, *p* = 0.02), suggesting a genetic susceptibility related to IL17A variants [40]. A review by Astarini et al. (2022) reported that IL17A polymorphisms contribute to hepatic steatosis, inflammation, and fibrosis, further supporting genetic involvement in MASLD [39].

As for metabolic-associated steatohepatitis (MASH), our study revealed that patients with MASH had a significantly increased susceptibility to presenting the IL 17F-*A7448G* variant genotype. Research suggests that IL17A/F plays a crucial role in the progression of MASH, promoting inflammation and liver damage. However, specific studies examining the IL 17F-*A7448G* variant as a risk factor for MASH are limited. IL17A has been shown to exacerbate hepatic steatosis and inflammation, contributing to the progression of MASH through pathways such as ERK1/2/p65 signaling, which promotes hepatocyte apoptosis [41]. In addition, IL17A influences liver disease by modulating immune responses, with increased levels of IL17A correlating with worsening liver disease in MASH and hepatocellular carcinoma (HCC) [38]. Even though these studies emphasize the importance of IL17A in MASH, further research is needed to precisely determine the impact of the IL17F-*A7448G* variant on susceptibility and progression of MASH in order to support our findings regarding the susceptibility of IL 17F-*A7448G* variant genotype in MASH population.

Although information about the association between IL17A/F and MASH polymorphisms is very limited, the association between TLR4 gene polymorphisms and the risk of MASH has been intensively researched. TLR4, activated by lipopolysaccharides (LPS) and other ligands, initiates signaling pathways that produce proinflammatory cytokines and chemokines, aggravating liver inflammation. Sun et al. (2023) showed that the eNAMPT/TLR4 inflammatory signaling pathway contributes significantly to MASLD severity and its progression to MASH and liver fibrosis, suggesting that neutralizing this pathway could reduce disease severity [42].

The *Asp299Gly* polymorphism impairs the ability of TLR4 to recruit MyD88 and TRIF adapters essential for signaling, thereby diminishing the inflammatory response to lipopolysaccharides (LPS), a crucial component of bacterial endotoxins. This reduction in the inflammatory response can lead to a less effective immune response, contributing to the chronic inflammation seen in MASH. Furthermore, the *Asp299Gly* variant has been shown to alter the overall function of TLR4 without changing its expression or binding ability, highlighting its specific impact on downstream signaling. Understanding these mechanisms underscores the variant’s potential role in modulating inflammatory pathways in MASH, suggesting it is a target for future research and possible therapeutic interventions [43,44].

On the other hand, research shows that the *Thr399Ile* polymorphism does not significantly alter TLR4 signaling or function, unlike the *Asp299Gly* variant, which considerably reduces the receptor response to lipopolysaccharides (LPS) [43]. Although TLR4 variants are present in patients with MASLD, the specific contribution of *Thr399Ile* to disease pathology needs to be clarified, as this variant does not appear to affect downstream signaling pathways significantly. In addition, TLR4-*Thr399Ile* has been associated with the presence of obesity, a comorbidity commonly found in patients with MASH, thus suggesting its indirect influence on liver disease via broader metabolic and inflammatory pathways [45].

In our research, we evaluated separately patients with simple steatosis (MAFL) or steatohepatitis (MASH) and patients with MASLD (both MASH and MAFL). We tried to establish the role of IL17 and TLR4 genes in their pathogenesis. We found a higher frequency of TLR4 polymorphism, *Asp299Gly*, and *Thr399Ile* in patients with MASH and MAFL. Univariate logistic regression analysis revealed significantly increased odds of MASH associated with a dominant model for TLR4-*Asp299Gly* (OR 3.52, *p* = 0.0338) and TLR4-*Thr399Ile* (OR 9.87, *p* = 0.0069). The results remained statistically significant after correction for multiple hypotheses testing for TLR4-*Asp299Gly* (*p* = 0.0338) and TLR4-*Thr399Ile* (OR 9.87, *p* = 0.0138). Regarding patients with MAFL, we found a statistically significant association only with the variant genotype G/A + A/A of the IL17A-*G197A* polymorphism. Analysis of the allelic model revealed significantly increased odds for MAFL in the presence of the *A* allele of IL17F-*A7488G* (*p* corrected = 0.0133) polymorphism. Also, increased odds of MASH were observed in the presence of the *A* allele of IL17A-*G197A* (*p* corrected = 0.008), Gly allele of TLR4-*Asp299Gly* (*p* corrected = 0.046), and *Ile* allele of TLR4-*Thr399Ile* (*p* corrected = 0.008) polymorphism.

If we analyzed patients with MASLD in general, our results suggested that after correction for multiple hypotheses testing, the increased odds in the dominant model were only for the TLR4-*Thr399Ile* (OR 5.78, *p* = 0.0274) polymorphism. Also, the TLR4-*Asp299Gly* (OR 2.91, *p* = 0.0288) and TLR4-*Thr399Ile* (OR 4.58, *p* = 0.0274) polymorphisms represent possible risk factors for MASLD in the allelic model.

A possible explanation for our results, which contradict other studies, could be the small size of the group of subjects from Romania, thus suggesting the polymorphism’s geographical and ethnic specificity. The fact that patients were included from only one medical center may introduce selection biases, as the population studied may not represent the broader community or other regions.

As for IL17 haplotyping analysis, it suggested that haplotype *A/A* (*A7488G*, *G197A*) represents a risk factor for MASLD. We found no TLR4 haplotypes as a risk factor for MASLD patients. This preliminary study found that patients with MASLD had significantly higher IL17A, IL17F, and TLR4 levels than controls. Analysis of IL17A, IL17F, and TLR4 levels according to the dominant model for these four polymorphisms investigated revealed that in controls, IL17A serum levels were significantly increased in carriers of the *A/G* + *G/G* genotypes compared with *A/A* genotype of IL 17F-*A7448G* gene polymorphism and also IL17F was significantly increased in carriers of the *Asp/Asp* compared to those with *Asp/Gly* + *Gly/Gly* genotypes of the TLR4-*Asp299Gly* gene polymorphism. In MASLD patients, we found higher IL17F levels in carriers of the *G/A + A/A* compared with carriers of the *G/G* genotype of the IL 17A-*G197A* polymorphism. Similar to our results, Wang et al. (2016) found that individuals carrying the TLR4 rs2737190-A/G genotype (vs. A/A) had a significantly increased risk of either clinical tuberculosis (OR: 1.31, 95% CI: 1.11–1.53) or sputum smear-positive tuberculosis (OR: 1.35, 95% CI: 1.13–1.61). The study also explored SNPs in IL-17A (rs3819024) and IL-17F (rs763780), which were found to have a relationship with tuberculosis prognosis [18].

The main limitation of our study is the relatively small number of patients due to the invasive method of diagnosing MASLD, which is why we consider further exhaustive research necessary. The small sample size can lead to potentially unreliable estimates of the regression model coefficients and larger confidence intervals. Consequently, the findings may not be generalizable to the target population, and the conclusions of this study should be interpreted with caution. The results of this study may have limited generalizability, taking into account that the present exploratory study was conducted in a single center. The study results might be influenced by confounding factors (the development of comorbidities and the potential effects of ongoing therapies) that were not adjusted for in the present study due to the small sample size. Other genes related to the immunity pathway and environmental factors should be considered to fully understand the potential role of the studied gene polymorphisms and MASLD’s etiology.

## 5. Conclusions

In summary, our study provided evidence that IL17F-G*7488A*, IL17A-*G197A*, TLR4-*Asp299Gly*, and TLR4-*Thr399Ile* influence the susceptibility to MASH. Also, the IL17A-*G197A* polymorphism influences the susceptibility to MAFL—moreover, IL 17A-*G197A*, TLR4-*Asp299Gly*, and TLR4-*Thr399Ile* influence susceptibility to MASLD. Polymorphisms in IL 17A, IL 17F, or TLR4 could influence serum IL 17A, IL 17F, and TLR4 levels. Further research with larger and more diverse groups is needed to validate our findings and assess the clinical relevance of these genetic variations in real-world settings.

## Figures and Tables

**Table 1:** PCR-RFLP genotyping conditions.

Genetic Variations	Sequences of the Primers	PCR Program	Restriction Enzyme	RFLP Method
IL17F-*A7488G*	Fw:5′-ACCAAGGCTGCTCTGTTTCT-3′ Rv:5′-GGTAAGGAGTGGCATTTCTA-3′	Initial Denaturation: 95 °C for 60 s 34 cycles Denaturation: 95 °C for 10 s Annealing: 54.1 °C for 20 s Extension: 72 °C for 20 s Final Extension: 72 °C for 5 min	*NlaIII*	*A7488* allele: 80.63 bp *G7488* allele: 143 bp
IL17A-G*197A*	Fw:5′-CAGAAGACCTACATGTTACT-3′ Rv:5′-GTAGCGCTATCGTCTCTCT-3′	Initial Denaturation: 94 °C for 60 s 35 cycles Denaturation: 95 °C for 10 s Annealing: 46.6 °C for 20 s Extension: 72 °C for 20 s Final Extension: 72 °C for 30 s	*XmnI*	*G197* allele: 344 bp *A197* allele: 213, 131 bp
TLR4-*Asp299Gly*	Fw:5′-GATTAGCATACTTAGACTACTACCTCCATG-3′ Rv:5′-GATCAACTTCTGAAAAAGCATTCCCAC-3′	Initial Denaturation: 94 °C for 60 s 35 cycles Denaturation: 95 °C for 10 s Annealing: 60 °C for 1′20 s Extension: 72 °C for 15 s Final Extension: 72 °C for 30 s	NcoI	*Asp299* allele: 188 bp *Gly299* allele: 168, 20 bp
TLR4-*Thr399Ile*	Fw:5′-GGTTGCTGTTCTCAAAGTGATTTTGGGAGAA-3′ Rv:5′-ACCTGAAGACTGGAGAGTGAGTTAAATGCT-3′	Initial Denaturation: 94 °C for 60 s 35 cycles Denaturation: 95 °C for 10 s Annealing: 67.4 °C for 32 s Extension: 72 °C for 20 s	*HinfI*	*Thr399* allele: 129 bp *Ile399* allele: 104, 25 bp

Fw—forward; Rv—reverse.

**Table 2:** Demographic and clinical features of controls and MASLD patients.

Variables	Controls (n_1_ = 39)	MAFL (n_2_ = 19)	MASH (n_3_ = 23)	*p* ^(a)^
Demographics				
Age (years)	52.9 (14.4)	53.6 (12.5)	56.4 (8.2)	0.584
Sex (male), n (%)	14 (35.9)	6 (31.6)	10 (43.5)	0.714
Smoking (yes), n (%)	10 (25.6)	9 (47.4)	9 (39.1)	0.227
Body measurements				
Height (cm)	167.1 (7.8)	168.4 (9.7)	169.0 (9.6)	0.679
Weight (kg)	76.9 (16.3)	91.1 (16.9)	92.5 (9.3)	0.00009 *
BMI (kg/m^2^)	27.5 (5.0)	31.9 (4.5)	32.6 (4.0)	0.0001 *
WC (cm)	86.9 (14.7)	96.9 (12.9)	99.9 (9.8)	0.001 *
SBP (mmHg)	130 (118 to 145)	130 (123 to 140)	140 (130 to 150)	0.1008
DBP (mmHg)	70 (63 to 80)	80 (76 to 83)	90 (80 to 93)	0.0004 *
Laboratory parameters				
FPG (mg/dL)	87 (79 to 97)	86 (85 to 99)	112 (96 to 150)	0.0008 *
HDL-C (mg/dL)	46.9 (12.0)	40.9 (10.4)	33.7 (9.7)	0.0001 *
LDL-C (mg/dL)	108.1 (46.2)	116.8 (36.9)	130.0 (41.3)	0.156
T Cholesterol (mg/dL)	176.6 (44.3)	188.1 (36.4)	206.7(33.7)	0.0194 *
TG (mg/dL)	101 (63 to 144)	167 (94 to 194)	185 (128 to 281)	0.0004 *
ALT (mg/dL)	24 (14 to 36)	27 (21 to 48)	37 (24 to 73)	0.0188 *
AST (mg/dL)	26 (20 to 37)	24 (20 to 33)	29 (24 to 56)	0.1586
ALP (U/I)	175.7 (71.5)	225.7 (75.9)	235.6 (92.6)	0.0086 *
GGT (U/I)	32 (21 to 45)	42 (30 to 58)	54 (39 to 93)	0.0076 *
TBil (mg/dL)	0.60 (0.50 to 0.90)	0.60 (0.50 to 0.60)	0.60 (0.50 to 0.90)	0.314
Frequent Comorbidities				
HTA, n (%)	15 (38.5)	9 (47.4)	16 (69.6)	0.0596
T2DM, n (%)	5 (12.8)	2 (10.5)	12 (52.2)	0.0006 *
Dyslipidemia, n (%)	0 (0.0)	7 (36.8)	12 (52.2)	<0.0001 *
Obesity, n (%)	0 (0.0)	4 (21.1)	12 (52.2)	<0.0001 *
CHD, n (%)	1 (2.6)	4 (21.1)	12 (52.2)	0.00001 *
AP, n (%)	2 (5.1)	3 (15.8)	9 (39.1)	0.0023 *
Histological characteristics				
Steatosis				<0.0001 *
0	39 (100.0)	0 (0.0)	0 (0.0)	
1	0 (0.0)	18 (94.7)	0 (0.0)	
2	0 (0.0)	1 (5.3)	18 (78.3)	
3	0 (0.0)	0 (0.0)	0 (0.0)	
Ballooning				<0.0001 *
0	39 (100.0)	16 (84.2)	7 (30.4)	
1	0 (0.0)	3 (15.8)	13 (56.5)	
2	0 (0.0)	0 (0.0)	3 (13.0)	
Lobular inflammation				<0.0001 *
0	39 (100.0)	13 (68.4)	5 (21.7)	
1	0 (0.0)	6 (31.6)	13 (56.5)	
2	0 (0.0)	0 (0.0)	4 (17.4)	
3	0 (0.0)	0 (0.0)	1 (4.3)	
Fibrosis				<0.0001 *
0	39 (100.0)	11 (57.9)	12 (52.2)	
1	0 (0.0)	6 (31.6)	6 (26.1)	
2	0 (0.0)	0 (0.0)	3 (13.0)	
3	0 (0.0)	2 (10.5)	2 (8.7)	
NAS score	-	1 (1 to 2)	4 (3 to 5)	

MAFL = metabolic-associated fatty liver, MASH = metabolic-associated steatohepatitis, T2DM = type 2 diabetes mellitus, CHD = chronic heart disease, AP = angina pectoris; data were summarized by mean (standard deviation); median (IQR) where IQR = Interquartile Range: (Q1, Q3), Q1 = first quartile; Q3 = third quartile ^(a)^ estimated from Welch’s ANOVA test, ANOVA test, Kruskal–Wallis test, Chi-squared test, exact Fisher’s test; * significant result: *p* < 0.05.

**Table 3:** Associations between studied SNPs and types of MASLD (MAFL and MASH).

		Controls (n_1_ = 39)	MAFL (n_2_ = 19)	MASH (n_3_ = 23)	Tests of Associations
*SNP*	Genotypes/Alleles	n (%)	n (%)	n (%)	*p* ^(a)^
IL17F-*A7448G*					0.0956
Co-dominant model	A/A	36 (92.3)	17 (89.5)	16 (69.6)	
	A/G	2 (5.1)	2 (10.5)	6(26.1)	
	G/G	1 (2.6)	0 (0.0)	1 (4.3)	
Dominant model	A/A	36 (92.3)	17 (89.5)	16 (69.6)	0.0703
	A/G +G/G	3 (7.7)	2 (10.5)	7 (30.4)	
Recessive model	A/A + A/G	38 (97.4)	19 (100)	22 (95.7)	1.000
	G/G	1 (2.6)	0 (0.0)	1 (4.3)	
Allelic model	A	74 (94.9)	36 (94.7)	38 (82.6)	0.063
	G	4 (5.1)	2 (5.3)	8 (17.4)	
IL17A-*G197A*					0.0004 *
Co-dominant model	G/G	34 (87.2)	11 (57.9)	9 (39.1)	
	G/A	3 (7.7)	5 (26.3)	12 (52.2)	
	A/A	2 (5.1)	3 (15.8)	2 (8.7)	
Dominant model	G/G	34 (87.2)	11 (57.9)	9 (39.1)	0.0003 *
	G/A + A/A	5 (12.8)	8 (42.1)	14 (60.9)	
Recessive model	G/G + G/A	37 (94.9)	16 (84.2)	21 (91.3)	0.3956
	A/A	2 (5.1)	3 (15.8)	2 (8.7)	
Allelic model	G	71 (91.0)	27 (71.1)	30 (65.2)	0.0010 *
	A	7 (9.0)	11 (28.9)	16 (34.8)	
TLR4-*Asp299Gly*					0.0691
Co-dominant model	Asp/Asp	32 (82.1)	13 (68.4)	13 (56.5)	
	Asp/Gly	7 (17.9)	4 (21.1)	9 (39.1)	
	Gly/Gly	0 (0.0)	2 (10.5)	1 (4.3)	
Dominant model	Asp/Asp	32 (82.1)	13 (68.4)	13 (56.5)	0.0925
	Asp/Gly + Gly/Gly	7 (17.9)	6 (31.6)	10 (43.5)	
Recessive model	Asp/Asp+ Asp/Gly	39 (100.0)	17 (89.5)	22 (95.7)	0.07821
	Gly/Gly	0 (0.0)	2 (10.5)	1 (4.3)	
Allelic model	Asp	71 (91.0)	30 (78.9)	35 (76.1)	0.054
	Gly	7 (9.0)	8 (21.1)	11 (23.9)	
TLR4-*Thr399Ile*					0.0151 *
Co-dominant model	Thr/Thr	37 (94.9)	17 (89.5)	15 (65.2)	
	Thr/Ile	1 (2.6)	1 (5.3)	6 (26.1)	
	Ile/Ile	1 (2.6)	1 (5.3)	2 (8.7)	
Dominant model	Thr/Thr	37 (94.9)	17 (89.5)	15 (65.2)	0.0054 *
	Thr/Ile + Ile/Ile	2 (5.2)	2 (10.6)	8 (34.8)	0.6790
Recessive model	Thr/Thr + Thr/Ile	38 (97.5)	18 (94.8)	21 (91.3)	
	Ile/Ile	1 (2.6)	1 (5.3)	2 (8.7)	
Allelic model	Thr	75 (96.2)	35 (92.2)	36 (78.3)	0.0060 *
	Ile	3 (3.8)	3 (7.9)	10 (21.7)	

SNPs = single nucleotide polymorphisms; MAFL = metabolic-associated fatty liver; MASH = metabolic-associated steatohepatitis; ^(a)^ estimated from Fisher’s exact test or Chi-square test; * results with statistical significance: *p* < 0.05.

**Table 4:** Results of genotypic analysis obtained from univariable multinomial logistic regression model differentiating controls from MAFL and MASH patients.

		MAFL Group (n = 19)			MASH Group (n = 23)		
*SNP*	Genotypes	OR (95% CI)	*p* ^(a)^	Corrected *p* ^(b)^	OR (95% CI)	*p* ^(a)^	Corrected *p* ^(b)^
IL17F-*A7448G*							
Dominant model	A/A	1 (Reference)			1 (Reference)		
	A/G +G/G	1.41 (0.22 to 9.25)	0.7192	0.7192	5.25 (1.20 to 22.95)	0.0276 *	0.0338 *
IL17A-*G197A*							
Dominant model	G/G	1 (Reference)			1 (Reference)		
	G/A + A/A	4.94 (1.34 to 18.28)	0.0166 *	0.0664	10.57 (3.01 to 37.19)	0.0002 *	0.0008 *
TLR4-*Asp299Gly*							
Dominant model	Asp/Asp	1 (Reference)			1 (Reference)		
	Asp/Gly + Gly/Gly	2.11 (0.59 to 7.49)	0.2480	0.4960	3.52 (1.10 to 11.23)	0.0338 *	0.0338 *
TLR4-*Thr399Ile*							
Dominant model	Thr/Thr	1 (Reference)			1 (Reference)		
	Thr/Ile + Ile/Ile	2.18 (0.28 to 16.78)	0.4555	0.6073	9.87 (1.87 to 51.97)	0.0069 *	0.0138 *

SNPs = single nucleotide polymorphisms; MAFL = metabolic-associated fatty liver, MASH = metabolic-associated steatohepatitis; OR = odds ratio; 95% CI = 95% confidence interval for OR; ^(a)^ estimated from univariable multinomial logistic regression model; ^(b)^ false-discovery-rate-corrected *p*-value for multiple hypotheses testing using the Benjamini–Hochberg (BH) correction; * results with statistical significance: *p* < 0.05.

**Table 5:** Genotypic and allelic associations between studied SNPs and odds of MASLD.

		MASLD Group (n = 42)		
SNP	Genotypes/Alleles	OR (95% CI)	*p* ^(a)^	Corrected *p* ^(b)^
IL17F-*A7448G*				
Dominant model	A/A	Reference	0.0755	0.0862
	A/G + G/G	3.27 (0.82 to 13.13)		
Allelic model	A	Reference	0.1250	0.1250
	G	2.50 (0.75 to 8.33)		
IL17A-*G197A*				
Dominant model	G/G	Reference	0.0001 *	0.0008 *
	G/A + A/A	7.48 (2.45 to 22.86)		
Allelic model	G	Reference	0.001 *	0.0040 *
	A	4.81 (1.95 to 11.83)		
TLR4-*Asp299Gly*				
Dominant model	Asp/Asp	Reference	0.0421 *	0.0561
	Asp/Gly + Gly/Gly	2.81 (1.01 to 7.86)		
Allelic model	Asp	Reference	0.0180 *	0.0288 *
	Gly	2.97 (1.17 to 7.51)		
TLR4-*Thr399Ile*				
Dominant model	Thr/Thr	Reference	0.0137 *	0.0274 *
	Thr/Ile + Ile/Ile	5.78 (1.18 to 28.35)		
Allelic model	Thr	Reference	0.0130 *	0.0274 *
	Ile	4.58 (1.25 to 16.74)		

SNPs = single nucleotide polymorphisms; MASLD = metabolic-associated steatotic liver disease, OR = odds ratio; 95% CI = 95% confidence interval for OR. ^(a)^ estimated from univariable binomial logistic regression model; ^(b)^ false-discovery-rate-corrected *p*-value for multiple hypotheses testing using the Benjamini–Hochberg (BH) correction; * results with statistical significance: *p* < 0.0.

**Table 6:** Haplotype analysis for testing the association between haplotypes of IL17 and TLR4 gene polymorphisms and risk of MASLD.

SNP1	SNP2	Haplotype Relative Frequencies		OR ^(a)^ (95% CI)	*p*
IL17F-*A7448G*	IL17A-*G197A*	Control Group	MASLD Group		
*A*	*G*	0.8590	0.5831	Reference	
*A*	*A*	0.0897	0.2979	3.72 (1.53 to 9.06)	0.0038 *
*G*	*G*	0.0513	0.0955	2.13 (0.59 to 7.66)	0.2449
*G*	*A*	NA	0.0235	NA	<0.001 *
Performance of haplotype-based GLM model: Statistics = 12.835, df = 3, *p* ^(b)^ = 0.0050					
TLR4-*Asp299Gly*	TLR4-*Thr399Ile*				
*Asp*	*Thr*	0.8718	0.6762	Reference	
*Asp*	*Ile*	0.0385	0.0976	2.32 (0.72 to 7.50)	0.1604
*Gly*	*Thr*	0.0897	0.1690	2.41 (0.90 to 6.46)	0.0801
*Gly*	*Ile*	NA	0.0572	NA	<0.001 *
Performance of haplotype-based GLM model: Statistics = 8.3466, df = 3, *p* ^(b)^ = 0.0394					

Note. Haplotypes estimated from the three variants; haplotypes frequencies inferred by haplo.stats R package; ^(b)^ *p*-values of the Global Score test for haplotype effect in additive model; NA = not available because of their relative frequency = 0 in control group; ^(a)^ OR = unadjusted odds ratio estimated from haplotype-based GLM regression without covariates; * result statistically significant (*p* < 0.05).

**Table 7:** Distributions of IL17 and TLR4 serum levels by genotypes of studied gene polymorphisms in the dominant model in controls and MASLD patients.

			IL17A		IL17F		TLR4	
Groups	SNP	Genotypes	Median (IQR)	*p*	Median (IQR)	*p*	Median (IQR)	*p*
Controls	IL17F-*A7488G*	*A/A* (n = 36)	67.8 (40.5 to 98.1)	0.0203 *	7.1 (5.3 to 10.0)	0.4932	1278.0 (957.9 to 1443.5)	0.0917
		*A/G +G/G* (n = 3)	399.5 (256.3 to 401.5)		19.9 (12.2 to 20.5)		1838.2 (1529.0 to 1926.3)	
	Il17A-*G197A*	*G/G* (n = 34)	76.6 (41.8 to 109.7)	0.8831	7.3 (5.3 to 12.9)	0.8665	1278.0 (998.9 to 1465.2)	0.8997
		*G/A + A/A* (n = 5)	64.9 (45.0 to 161.2)		6.9 (5.8 to 8.5)		1348.5 (1004.2 to 1448.1)	
	TLR4-*Asp299Gly*	*Asp/Asp* (n = 32)	73.4 (43.9 to 129.0)	0.8692	8.3 (5.7 to 18.5)	0.0157 *	1316 (989.1 to 1475.1)	0.3797
		*Asp/Gly + Gly/Gly* (n = 7)	77.1 (45.9 to 92.3)		4.3 (3.9 to 4.6)		1217.8 (1035.3 to 1275.9)	
	TLR4-*Thr399Ile*	*Thr/Thr* (n = 37)	70.6 (40.8 to 118.3)	0.4078	6.9 (5.2 to 13.4)	0.7744	1259.3 (962.7 to 1448.1)	0.3083
		*Thr/Ile + Ile/Ile* (n = 2)	93.6 (90.8 to 96.5)		7.8 (7.5 to 8.1)		1426.3 (1398.9 to 1453.8)	
MASLD	IL17F-*A7488G*	*A/A* (n = 33)	143.6 (87.9 to 222.6)	0.7244	19.3 (8.3 to 27.2)	0.4165	1815.3 (1533.2 to 1935.7)	0.7709
		*A/G + G/G* (n = 9)	105.5 (95.9 to 122.6)		8.4 (6.8 to 16.1)		1894.2 (1446.1 to 2097.5)	
	Il17A-*G197A*	*G/G* (n = 20)	156.2 (83.1 to 227.3)	0.3917	8.3 (6.2 to 26.7)	0.0343 *	1845.4 (1440.9 to 1984.4)	0.8898
		*G/A + A/A* (n = 22)	103.7 (91.0 to 168.3)		21.4 (9.2 to 34.6)		1790.5 (1693.5 to 1939.8)	
	TLR4-*Asp299Gly*	*Asp/Asp* (n = 26)	122.8 (94.9 to 218.5)	0.4924	18.6 (47.5 to 26.9)	0.9380	1801.9 (1453.3 to 1940.3)	0.8969
		*Asp/Gly + Gly/Gly* (n = 16)	104.6 (87.5 to 190.3)		12.8 (7.7 to 27.4)		1820.5 (1488.6 to 2043.0)	
	TLR4-*Thr399Ile*	*Thr/Thr* (n = 32)	108.9 (89.3 to 199.1)	0.6577	11.7 (7.3 to 27.8)	0.8477	1753.2 (1488.6 to 1949.2)	0.7341
		*Thr/Ile + Ile/Ile* (n = 10)	110.5 (95.9 to 218.5)		19.8 (11.3 to 25.7)		1901.4 (1511.9 to 2002.1)	

MASLD = metabolic-associated steatotic liver disease, IQR = Interquartile Range: (Q1, Q3), Q1 = first quartile; Q3 = third quartile; all interquartile range are inclusive intervals; * result statistically significant (*p* < 0.05).

## Data Availability

The raw data used in this study can be obtained upon reasonable request from Lucia Maria Procopciuc (lprocopciuc@umfcluj.ro) and Sorina Cezara Coste (secara.sorina@umfcluj.ro; secara.sorina@yahoo.com).

## References

[1] Rinella M.E., Lazarus J.V., Ratziu V., Francque S.M., Sanyal A.J., Kanwal F., Romero D., Abdelmalek M.F., Anstee Q.M., Arab J.P. (2023). A Multisociety Delphi Consensus Statement on New Fatty Liver Disease Nomenclature. Hepatology.

[2] Riazi K., Azhari H., Charette J.H., Underwood F.E., King J.A., Afshar E.E., Swain M.G., Congly S.E., Kaplan G.G., Shaheen A.-A. (2022). The Prevalence and Incidence of NAFLD Worldwide: A Systematic Review and Meta-Analysis. Lancet Gastroenterol. Hepatol..

[3] Coste S.C., Popovici I., Stefan A.M., Breaban I., Sitar Taut A., Tarmure Sarlea S., Cozma A., Sampelean D., Orasan O.H., Negrean V. (2019). Immune and Inflammatory Pathways in Non-Alcoholic Steatohepatitis (NASH). An Update. J. Mind Med. Sci..

[4] Ballestri S., Zona S., Targher G., Romagnoli D., Baldelli E., Nascimbeni F., Roverato A., Guaraldi G., Lonardo A. (2016). Nonalcoholic Fatty Liver Disease Is Associated with an Almost Twofold Increased Risk of Incident Type 2 Diabetes and Metabolic Syndrome. Evidence from a Systematic Review and Meta-Analysis. J. Gastroenterol. Hepatol..

[5] Kawai T., Akira S. (2010). The Role of Pattern-Recognition Receptors in Innate Immunity: Update on Toll-like Receptors. Nat. Immunol..

[6] Rivera C.A., Adegboyega P., van Rooijen N., Tagalicud A., Allman M., Wallace M. (2007). Toll-like Receptor-4 Signaling and Kupffer Cells Play Pivotal Roles in the Pathogenesis of Non-Alcoholic Steatohepatitis. J. Hepatol..

[7] Hepatic TLR4 Signaling in Obese NAFLD|American Journal of Physiology-Gastrointestinal and Liver Physiology. https://journals.physiology.org/doi/full/10.1152/ajpgi.00304.2014.

[8] Cengiz M., Ozenirler S., Elbeg S. (2015). Role of Serum Toll-like Receptors 2 and 4 in Non-alcoholic Steatohepatitis and Liver Fibrosis. J. Gastroenterol. Hepatol..

[9] Abou El-Khier N.T., Elhammady D., Arafa M.M., Shahin D., Eladl E., Abousamra N.K., Sharaf-Eldeen O., Shaker G., Esmael M.E. (2018). Th17 and IL-17 as Predictors of Hepatic Inflammation in Patients with Chronic Hepatitis C Virus Infection and Treated with Direct Antiviral Therapy. Egypt. J. Immunol..

[10] Giles D.A., Moreno-Fernandez M.E., Stankiewicz T.E., Cappelletti M., Huppert S.S., Iwakura Y., Dong C., Shanmukhappa S.K., Divanovic S. (2016). Regulation of Inflammation by IL-17A and IL-17F Modulates Non-Alcoholic Fatty Liver Disease Pathogenesis. PLoS ONE.

[11] Ma H.-Y., Xu J., Liu X., Zhu Y., Gao B., Karin M., Tsukamoto H., Jeste D.V., Grant I., Roberts A.J. (2016). The Role of IL-17 Signaling in Regulation of the Liver-Brain Axis and Intestinal Permeability in Alcoholic Liver Disease. Curr. Pathobiol. Rep..

[12] Long Z.-W., Yu H.-M., Wang Y.-N., Liu D., Chen Y.-Z., Zhao Y.-X., Bai L. (2015). Association of IL-17 Polymorphisms with Gastric Cancer Risk in Asian Populations. World J. Gastroenterol. WJG.

[13] Tang Y., Bian Z., Zhao L., Liu Y., Liang S., Wang Q., Han X., Peng Y., Chen X., Shen L. (2011). Interleukin-17 Exacerbates Hepatic Steatosis and Inflammation in Non-Alcoholic Fatty Liver Disease. Clin. Exp. Immunol..

[14] Rock F.L., Hardiman G., Timans J.C., Kastelein R.A., Bazan J.F. (1998). A Family of Human Receptors Structurally Related to Drosophila Toll. Proc. Natl. Acad. Sci. USA.

[15] Arbour N.C., Lorenz E., Schutte B.C., Zabner J., Kline J.N., Jones M., Frees K., Watt J.L., Schwartz D.A. (2000). TLR4 Mutations Are Associated with Endotoxin Hyporesponsiveness in Humans. Nat. Genet..

[16] Olveira A., Augustin S., Benlloch S., Ampuero J., Suárez-Pérez J.A., Armesto S., Vilarrasa E., Belinchón-Romero I., Herranz P., Crespo J. (2023). The Essential Role of IL-17 as the Pathogenetic Link between Psoriasis and Metabolic-Associated Fatty Liver Disease. Life.

[17] Wang J., Liu Y., Xie L., Li S., Qin X. (2016). Association of IL-17A and IL-17F Gene Polymorphisms with Chronic Hepatitis B and Hepatitis B Virus-Related Liver Cirrhosis in a Chinese Population: A Case-Control Study. Clin. Res. Hepatol. Gastroenterol..

[18] Wang M., Xu G., Lü L., Xu K., Chen Y., Pan H., Burstrom B., Burstrom K., Wang J. (2016). Genetic Polymorphisms of IL-17A, IL-17F, TLR4 and miR-146a in Association with the Risk of Pulmonary Tuberculosis. Sci. Rep..

[19] Simundic T., Jelakovic B., Dzumhur A., Turk T., Sahinovic I., Dobrosevic B., Takac B., Barbic J. (2017). Interleukin 17A and Toll-like Receptor 4 in Patients with Arterial Hypertension. Kidney Blood Press. Res..

[20] Chalasani N., Younossi Z., Lavine J.E., Diehl A.M., Brunt E.M., Cusi K., Charlton M., Sanyal A.J. (2012). The Diagnosis and Management of Non-Alcoholic Fatty Liver Disease: Practice Guideline by the American Association for the Study of Liver Diseases, American College of Gastroenterology, and the American Gastroenterological Association. Hepatol. Baltim. Md.

[21] Bahadori M., Zarei S., Zarnani A.H., Zarei O., Idali F., Hadavi R., Jeddi-Tehrani M. (2014). IL-6, IL-10 and IL-17 Gene Polymorphisms in Iranian Women with Recurrent Miscarriage. Iran. J. Immunol..

[22] Wu X., Zeng Z., Chen B., Yu J., Xue L., Hao Y., Chen M., Sung J.J.Y., Hu P. (2010). Association between Polymorphisms in Interleukin-17A and Interleukin-17F Genes and Risks of Gastric Cancer. Int. J. Cancer Wiley Online Libr..

[23] Dhifallah I., Lachheb J., Houman M., Hamzaoui K. (2009). Toll-like-Receptor Gene Polymorphisms in a Tunisian Population with Behçet’s Disease. Clin. Exp. Rheumatol..

[24] Warnes G., Gorjanc G., Leisch F., Man M., Warnes G., Gorjanc G., Leisch F., Man M. (2021). Genetics: Population Genetics. R Package Version 1.3.8.1.3. https://cran.r-project.org/web/packages/genetics/index.html.

[25] Moreno V., Gonzalez J.R., Pelegri D., Moreno V., Gonzalez J., Pelegri D. (2022). SNPassoc: SNPs-Based Whole Genome Association Studies R Package Version 2.1.0. https://CRAN.R-Project.Org/package=SNPassoc.

[26] Venables W.N., Ripley B.D., Venables W.N., Ripley B.D. (2002). Modern Applied Statistics with S.

[27] Sinnwell J.P., Schaid D.J. (2024). haplo.Stats: Statistical Analysis of Haplotypes with Traits and Covariates When Linkage Phase Is Ambiguous_. R Package Version 1.9.5.1. https://CRAN.R-project.org/package=haplo.stats.

[28] Adult Overweight and Obesity|Overweight & Obesity|CDC. https://www.cdc.gov/healthy-weight-growth/food-activity/overweight-obesity-impacts-health.html.

[29] Jiang Z.-S., Wang S.-X., Jia H.-X., Wang J., Liu Y.-T. (2013). Association of Toll-Like Receptor 4 Polymorphisms with Type 2 Diabetes Mellitus. Inflammation.

[30] Kolz M., Baumert J., Müller M., Khuseyinova N., Klopp N., Thorand B., Meisinger C., Herder C., Koenig W., Illig T. (2008). Association between Variations in the TLR4gene and Incident Type 2 Diabetes Is Modified by the Ratio of Total Cholesterol to HDL-Cholesterol. BMC Med. Genet..

[31] Maldonado-Bernal C., Trejo-de la O.A., Sánchez-Contreras M.E., Wacher-Rodarte N., Torres J., Cruz M. (2011). Low Frequency of Toll-like Receptors 2 and 4 Gene Polymorphisms in Mexican Patients and Their Association with Type 2 Diabetes. Int. J. Immunogenet..

[32] Illig T., Bongardt F., Schöpfer A., Holle R., Müller S., Rathmann W., Koenig W., Meisinger C., Wichmann H.-E., Kolb H. (2003). The Endotoxin Receptor TLR4 Polymorphism Is Not Associated With Diabetes or Components of the Metabolic Syndrome. Diabetes.

[33] Cuda C., Badawi A., Karmali M., El-Sohemy A. (2011). Polymorphisms in Toll-like Receptor 4 Are Associated with Factors of the Metabolic Syndrome and Modify the Association between Dietary Saturated Fat and Fasting High-Density Lipoprotein Cholesterol. Metabolism.

[34] Zhao X., Zheng J., Wang J., Li B., Huang W. (2024). Inhibition of Hyperglycemia and Hyperlipidemia by Blocking Toll-like Receptor 4: Comparison of Wild-Type and Toll-like Receptor 4 Gene Knockout Mice on Obesity and Diabetes Modeling. Biology.

[35] Steinhardt A.P., Aranguren F., Tellechea M.L., Rosso L.A.G., Brites F.D., Martínez-Larrad M.T., Serrano-Ríos M., Frechtel G.D., Taverna M.J. (2010). A Functional Nonsynonymous Toll-like Receptor 4 Gene Polymorphism Is Associated with Metabolic Syndrome, Surrogates of Insulin Resistance, and Syndromes of Lipid Accumulation. Metabolism.

[36] Kiziltas S., Ata P., Colak Y., Mesçi B., Senates E., Enc F., Ulasoglu C., Tuncer I., Oguz A. (2014). TLR4 Gene Polymorphism in Patients with Nonalcoholic Fatty Liver Disease in Comparison to Healthy Controls. Metab. Syndr. Relat. Disord..

[37] Nguyen J., Jiao J., Smoot K., Watt G.P., Zhao C., Song X., Stevenson H.L., McCormick J.B., Fisher-Hoch S.P., Zhang J. (2018). Toll-like Receptor 4: A Target for Chemoprevention of Hepatocellular Carcinoma in Obesity and Steatohepatitis. Oncotarget.

[38] Gomes A.L., Teijeiro A., Burén S., Tummala K.S., Yilmaz M., Waisman A., Theurillat J.-P., Perna C., Djouder N. (2016). Metabolic Inflammation-Associated IL-17A Causes Non-Alcoholic Steatohepatitis and Hepatocellular Carcinoma. Cancer Cell.

[39] Astarini F.D., Ratnasari N., Wasityastuti W. (2022). Update on Non-Alcoholic Fatty Liver Disease-Associated Single Nucleotide Polymorphisms and Their Involvement in Liver Steatosis, Inflammation, and Fibrosis: A Narrative Review. Iran. Biomed. J..

[40] Akbulut U.E., Emeksiz H.C., Citli S., Cebi A.H., Korkmaz H.A.A., Baki G. (2019). IL-17A, MCP-1, CCR-2, and ABCA1 polymorphisms in children with non-alcoholic fatty liver disease. J. Pediatr. Versão Em Port..

[41] Pan Y., Ren X., Zhang Y., Lv J., Zeng Q., Zhang H., Yu Z. (2019). IL-17A-Mediated ERK1/2/P65 Signaling Pathway Is Associated with Cell Apoptosis after Non-Alcoholic Steatohepatitis. IUBMB Life.

[42] Sun B.L., Sun X., Kempf C.L., Song J.H., Casanova N.G., Camp S.M., Reyes Hernon V., Fallon M., Bime C., Martin D.R. (2023). Involvement of eNAMPT/TLR4 Inflammatory Signaling in Progression of Non-Alcoholic Fatty Liver Disease, Steatohepatitis, and Fibrosis. FASEB J. Off. Publ. Fed. Am. Soc. Exp. Biol..

[43] Long H., O’Connor B.P., Zemans R.L., Zhou X., Yang I.V., Schwartz D.A. (2014). The Toll-Like Receptor 4 Polymorphism Asp299Gly but Not Thr399Ile Influences TLR4 Signaling and Function. PLoS ONE.

[44] Figueroa L., Xiong Y., Song C., Piao W., Vogel S.N., Medvedev A.E. (2012). The Asp299Gly Polymorphism Alters TLR4 Signaling by Interfering with Recruitment of MyD88 and TRIF. J. Immunol..

[45] Weyrich P., Staiger H., Stančáková A., Machicao F., Machann J., Schick F., Stefan N., Kuusisto J., Laakso M., Schäfer S. (2010). The D299G/T399I Toll-Like Receptor 4 Variant Associates with Body and Liver Fat: Results from the TULIP and METSIM Studies. PLoS ONE.

